# Disparities in Lung Cancer: A Targeted Literature Review Examining Lung Cancer Screening, Diagnosis, Treatment, and Survival Outcomes in the United States

**DOI:** 10.1007/s40615-023-01625-2

**Published:** 2023-05-19

**Authors:** Lisa L. Dwyer, Pratyusha Vadagam, Julie Vanderpoel, Carol Cohen, Benjamin Lewing, Joseph Tkacz

**Affiliations:** 1 Real World Value & Evidence, Janssen Scientific Affairs, LLC, 1125 Trenton-Harbourton Road, Titusville, NJ 08560 USA; 2 Inovalon, Bowie, MD USA

**Keywords:** Lung cancer, Social determinants of health, Health disparities, African American, Black, Health equity

## Abstract

**Background:**

Although incidence and mortality of lung cancer have been decreasing, health disparities persist among historically marginalized Black, Hispanic, and Asian populations. A targeted literature review was performed to collate the evidence of health disparities among these historically marginalized patients with lung cancer in the U.S.

**Methods:**

Articles eligible for review included 1) indexed in PubMed®, 2) English language, 3) U.S. patients only, 4) real-world evidence studies, and 5) publications between January 1, 2018, and November 8, 2021.

**Results:**

Of 94 articles meeting selection criteria, 49 publications were selected, encompassing patient data predominantly between 2004 and 2016. Black patients were shown to develop lung cancer at an earlier age and were more likely to present with advanced-stage disease compared to White patients. Black patients were less likely to be eligible for/receive lung cancer screening, genetic testing for mutations, high-cost and systemic treatments, and surgical intervention compared to White patients. Disparities were also detected in survival, where Hispanic and Asian patients had lower mortality risks compared to White patients. Literature on survival outcomes between Black and White patients was inconclusive. Disparities related to sex, rurality, social support, socioeconomic status, education level, and insurance type were observed.

**Conclusions:**

Health disparities within the lung cancer population begin with initial screening and continue through survival outcomes, with reports persisting well into the latter portion of the past decade. These findings should serve as a call to action, raising awareness of persistent and ongoing inequities, particularly for marginalized populations.

**Supplementary Information:**

The online version contains supplementary material available at 10.1007/s40615-023-01625-2.

## Introduction

Lung (and bronchus) cancer is the second most common cancer diagnosis in the United States (U.S.), and the most common cause of cancer deaths with 127,070 deaths estimated for 2023 [1–3]. The reasons for poor survival are multifaceted and are related to tobacco use, late-stage diagnosis, and underutilization of newer and effective treatments [3, 4]. Public health education and implementation of tobacco control policies have resulted in decreased lung cancer deaths since the early 1980’s for men and the mid-2000’s for women, which also highlight a gender disparity in smoking cessation [3]. Lung cancer screening eligibility and access for at-risk populations have improved early detection [5, 6]. This has mitigated late-stage diagnosis with earlier treatment intervention [6, 7]. Genetic mutation testing, advances in the field of tumor biology, and novel targeted therapies have improved survival outcomes [8–10]. Despite these crucial medical advances, lung cancer disparities exist among marginalized racial and ethnic groups [11, 12], particularly Black as well as Hispanic, Asian/Pacific Islanders, and Indigenous patients [11]. Compared to White patients, Black patients were 16% less likely to be diagnosed early with lung cancer, 19% less likely to receive surgical treatment, and 7% more likely to receive no treatment [12]. Disparities in these outcomes have also been observed for Latinos, Asian Americans, and Indigenous people [12, 13]. Race and ethnicity are only some of the drivers of health disparities. Individuals residing in rural communities experience higher death rates from lung cancer due, in part, to poverty and health risk behaviors, while individuals with lower socioeconomic status also have higher incidence rates and poorer outcomes [14, 15].

Addressing health disparities, operationally defined as preventable differences in health outcomes and opportunities for optimal health among “socially disadvantaged groups”, is a mission of the U.S. public health community, as demonstrated by initiatives of Healthy People 2020 and 2030 and the National Partnership for Action to End Health Disparities [16–18]. Understanding the interplay of disparities within racially marginalized populations that historically have been underserved is critical to reduce population health inequities and advance social justice. Indeed, many private sector companies within the healthcare industry, as well as non-profit health agencies, have also made commitments and investments in health equity [19, 20] and Diversity, Equity, and Inclusion programs [21].

Prior literature reviews on lung cancer disparities [22, 23] and those identified from the current search [24–26] include limited topics such as disease incidence in diverse populations, racial and socioeconomic disparities in screening and treatment, and etiological factors such as tobacco smoking and environmental/occupational exposures that contribute to observed disparities [22–26]. However, since prior lung cancer reviews focus only on specific topics, they each underestimate the extent of disparities across the clinical care continuum. As such, we sought to summarize more current data in a singular comprehensive review by collating evidence of the totality of health disparities. This includes sociodemographic, socioeconomic, neighborhood, and environmental factors in the multiple settings of screening, diagnosis, treatment, and survival outcomes currently experienced by Black and other marginalized racial and ethnic populations with lung cancer. To accomplish this encompassing goal, we utilized a targeted literature review (TLR), also known as a focused review, of clinically relevant information to better inform a diverse range of professionals─such as primary care practitioners, social workers, hospital administrators, and health policy experts─concerned about racial disparities.

## Methods

A TLR was undertaken to provide a focused review of disparities observed along the lung cancer patient journey. The article search was limited to the PubMed® Database that supports Medline and a variety of health equity/policy journals. Grey literature provided important statistics, facts, and other data offering a comprehensive view of the topic for the Introduction and Discussion.

The first phase of the review used a three-tiered strategy to identify the focus areas of racially marginalized populations, sociodemographics, socioeconomics, and undertreatment. The goal was to better understand the relative contributions of these sub-topics to the overall article pool (Table 1). Advanced PubMed® searches were conducted using search term strings limited to the article title to maximize the relevance of articles. In cases of low article yield, the search was repeated using the title and abstract. Eligible articles were required to be written in English, include U.S. patients only, and be published between January 1, 2018 and November 8, 2021. These dates were chosen to allow for the lag-time associated with data accrual in real-world databases to retrieve the most current information available. A preliminary review of published articles prior to 2018 yielded less recent findings that dated back to the 1990’s.

**Table 1:** Tiered approach to article selection

Step	Search string
1. Initial pass-through of the literature to examine general size and scope of published articles focused on health disparities in lung cancer, specifically as it relates to minority health	("Lung neoplasm" OR “Lung cancer” OR “lung carcinoma” OR “non-small-cell lung carcinoma” OR “cancer(s) of the lung”) AND (“race” OR “Black” OR “African American” OR “disparities” OR “minority” OR “minority health” OR “Afro-Caribbean”)
2. Initial pass-through of the literature to examine general size and scope of published articles focused on health disparities in lung cancer, specifically as it relates to other sociodemographic and socioeconomic factors	("Lung neoplasm" OR “Lung cancer” OR “lung carcinoma” OR “non-small-cell lung carcinoma” OR “cancer(s) of the lung”) AND (“social determinants of health” OR “demographics” OR “sociodemographic” OR “low-income” OR “segregated” OR “residential segregation” OR “poverty”)
3. Initial pass-through of the literature to examine general size and scope of published articles focused on health disparities in lung cancer, specifically as it relates to undertreatment	("Lung neoplasm" OR “Lung cancer” OR “lung carcinoma” OR “non-small-cell lung carcinoma” OR “cancer(s) of the lung”) AND (“under-treatment” OR “undertreated” OR “suboptimal care” OR “low-quality care” OR “under-resourced” OR “urban centers” OR “urban communities”)

An emphasis was placed on identifying real-world evidence (RWE) studies, other targeted reviews, systematic reviews, and meta-analyses. RWE studies, frequently reported in oncology literature, capture data from routine clinical practice and provide an understanding of the effectiveness of treatments in the general population [27]. Furthermore, RWE studies can prove useful in demonstrating the treatment experience of patients who are often excluded (because of exclusion criteria) or underrepresented in randomized controlled trials [27]. This current TLR predominately includes data from a variety of large national, state, and local/hospital cancer registries, electronic health records, and other administrative databases.

Article abstracts were reviewed and judged for relevance by five research reviewers (LD, JV, CC, BL, JT) based upon three criteria: (1) clinical focus (screening, diagnosis, treatments, survival, and other clinically relevant topics); (2) data sources (national, state, and local database resources); and (3) patient social determinants of health and physician training and attitudes. For topics that were frequently captured (e.g., lung cancer screening), emphasis was placed on more recent publications with larger samples, as was the inclusion of Hispanic patients and other racially marginalized groups (Asian/Pacific Islander, American Indian, etc.). A total of 49 articles were selected for inclusion.

Following article selection, a template based on the PICOTS [28] (i.e., patient/population, intervention/assessment, comparison, outcome(s), time, study design) was used to extract applicable information from each article. Article review and data extraction were shared amongst three research reviewers (CC, BL, JT).

## Results

**Fig. 1:**
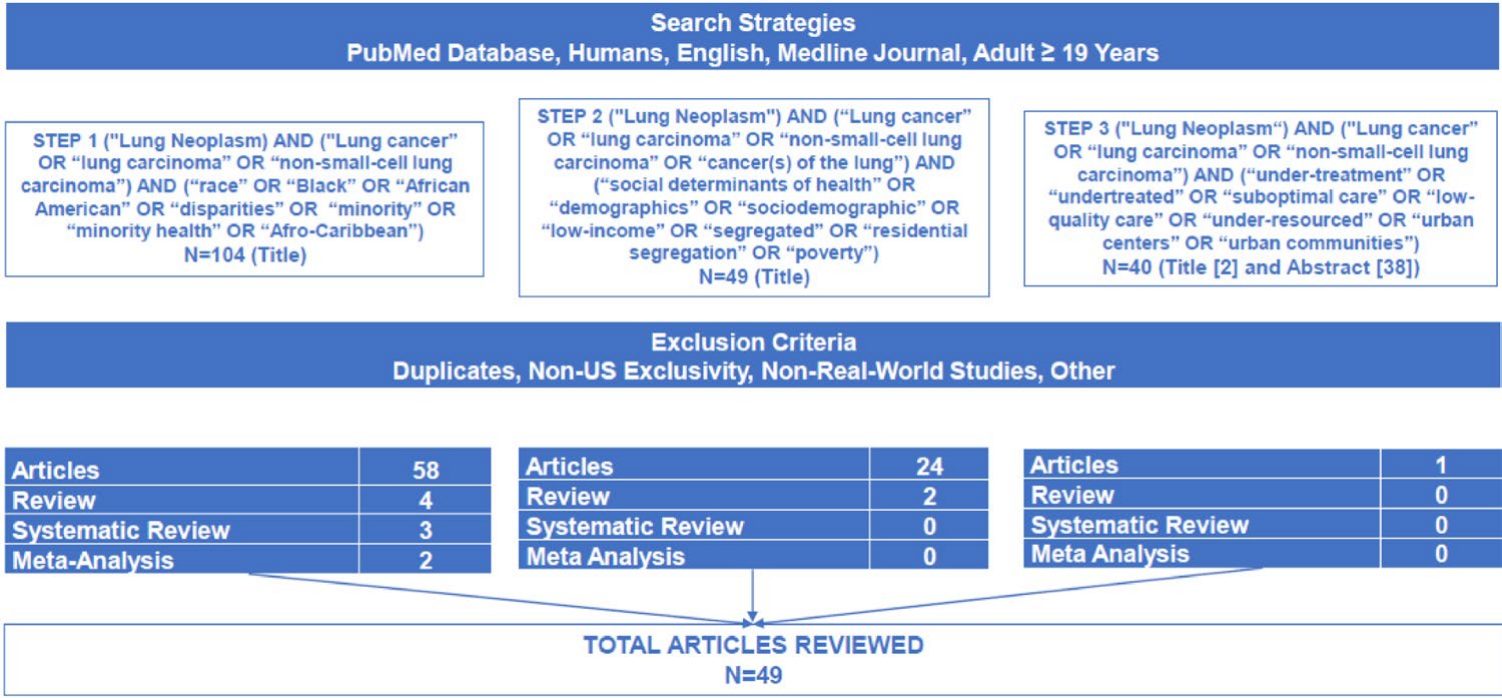
Article search results

The initial PubMed® search returned 104, 49, and 40 articles, respectively, for each of the three focus areas identified as Steps 1 through 3 (Fig. 1). After applying all exclusion criteria, 67, 26, and 1 article(s) remained for the respective search groups. Abstracts from all remaining articles were reviewed in detail by the five-person review team, which resulted in the exclusion of additional articles due to redundancies in topics stated above and analyses that included outdated measurement windows. A total of 49 articles were selected for inclusion in the final review, reflecting patient data predominately from 2004 to 2016.

The final article extraction and summary table is presented in Supplementary Table 1 and includes the key elements from each of the 49 articles selected. Reviewed articles included retrospective and prospective studies, as well as select reviews, systematic reviews, and meta-analyses. Common retrospective data sources included the Surveillance, Epidemiology, and End Results (SEER) database (*n* = 11), databases maintained by the Centers for Disease Control and Prevention (CDC) (*n* = 6), the U.S. National Cancer Database (NCD) (*n* = 6), and select state and hospital databases. Reviewed studies included patients with various lung cancer types and stages, including non-small cell lung carcinoma (NSCLC) and metastatic lung cancer, although numerous studies did not specify this level of detail. Most articles assessed patient race and ethnicity (*n* = 43). The primary social determinants of health and sociodemographic measures observed were income/socioeconomic status/poverty level (*n* = 14), rurality (*n* = 9), health insurance status (*n* = 7), sex (*n* = 7), and education level (*n* = 2). The reviewed articles covered a variety of clinical topics, including lung cancer screening (*n* = 16), genetic testing and mutation rates (*N* = 5), type and timeliness of lung cancer treatment (*n* = 17), and survival (*n* = 3). Additional topics included hospice utilization, smoking, and occupational risks. Details of the findings are summarized below.

### Overview of the Demographics, Incidence, and Etiological Factors of Lung Cancer in Minoritized Groups

Haddad et al.’s [24] review article on lung cancer screening provided an overview of lung cancer demographics in the U.S. Black males were shown to have the highest rate of lung cancer mortality among all racial groups, in addition to having the highest age-adjusted lung cancer incidence among both smoker and never-smoker sub-groups. Black patients developed lung cancer at an earlier age than White patients (median age 67 vs. 70) and were likely to present with advanced-stage disease (53% vs. 49%). Further, Haddad et al.’s review indicated that Hispanic and American Indian/Alaskan Native communities are underrepresented in published literature, although Hispanics have been shown to have lower smoking prevalence and lung cancer mortality compared to both Black and White individuals. The prevalence of cigarette smoking was greatest among American Indian/Alaskan Native communities, despite having both lower incidence and mortality rates compared to Black and White communities. HIV-positive patients are also at high risk, with lung cancer incidence three times higher than that of the general population. Ryan [26] noted numerous etiological factors contributing to lung cancer disparities, including smoking (i.e., screening age, dose, duration, cessation, menthol, genetics, and metabolism), environment (i.e., body mass index, alcohol consumption, radon, pollution, and geographic location), early detection (i.e., screening eligibility, screening uptake, and biomarkers), societal factors (stage at presentation, insurance status, belief systems, socioeconomic status, access to care, use of care, and health literacy), and biology (i.e., genetics and metabolism, transcriptomics, and biomarkers). Disparity in cancer risk by neighborhood deprivation was detected among current and former smokers in a cohort study of patients residing in the southeastern U.S., although it was not associated with increased lung cancer risk by sex or race [29]. Using nationwide county-level data, Houston et al. [30] observed higher lung cancer incidence for Non-Hispanic Black patients living in metropolitan and non-adjacent counties compared with Non-Hispanic White patients. This disparity increased the further the counties were from the metropolitan areas. Poor oral health was shown to be associated with increased lung cancer risk among Black individuals as well [31]. This likely reflects smoking and/or limited access to regular dental care, suggesting poor oral health among socioeconomically disadvantaged populations.

### Lung Cancer Screening

Lung cancer screening was one of the most observed topics in the health disparities literature, accounting for 16 articles [24, 32–46]. Lung cancer screening allows detection at an early stage when interventions can be more effective. Haddad et al. [24] noted that disparities in lung cancer screening were associated with race and ethnicity, rurality, environment (i.e., radon), occupational exposure (i.e., asbestos), HIV Infection, access to care, and patient-level barriers (i.e., socioeconomic status, geographic location, insurance).

In general, studies largely support that Black patients are more likely to be diagnosed with lung cancer at a later stage compared to White patients. Annangi et al. [32] showed the differences between stage III and stage IV diagnoses between White and Black patients to be 23.4% vs. 25.9% and 42.9% vs. 46.0%, respectively (*p* < 0.05). Lake et al. [36] conducted a historical cohort study of patients referred to an urban academic medical center. Black patients had lower odds of receiving low-dose computed tomography screening compared to White patients, lower rates of returning for annual screenings, and higher rates of loss to follow-up. The National Lung Screening Trial (NLST) identified being female, married/living as married, and being a former smoker as predictors of follow-up after a positive lung cancer screening [37]. White patients compared with Black patients had statistically higher rates of follow-up care after screening tests (89.6% vs. 82.8%; *p* < 0.05).

Using the SEER database and adjusting for demographic, socioeconomic, and facility characteristics, Black patients with squamous cell NSCLC were about half as likely to receive a positron emission tomography (PET), and Hispanic patients were about two-thirds as likely to receive a PET scan compared with Non-Hispanic White patients [38]. Interestingly, numerous studies also show that Black individuals were less likely to qualify for lung cancer screening eligibility compared to White individuals [34, 42, 44], due in part to lower tobacco exposure and younger age at time of diagnosis [33]. Japuntich et al. [35] showed that Non-Black patients were 90% more likely to meet the U.S. Preventive Services Taskforce (USPSTF) criteria for lung cancer screening compared to Black patients, while Pasquinelli et al. [40] found the PLCOm2012 model (Prostate, Lung, Colorectal and Ovarian Cancer Screening Trial) performed better in the identification of Black ever-smokers eligible for lung cancer screening.

One study examined the impact of rurality on lung cancer screening [45]. Using the Census block group and county-level data from Missouri and Illinois, this study demonstrated that, compared with 41% of nonmetropolitan residents, approximately 98% of metropolitan residents had access to screening. Multivariable analyses did not reveal an association between geographic screening access and lung cancer mortality, suggesting lung cancer mortality in rural regions is multifactorial and cannot be explained by access to screening alone. These rural areas have higher smoking prevalence than urban areas.

### Lung Cancer Genetic Testing and Mutation Rates

Disparities in genetic testing and mutational rates served as the primary focal point of five articles [47–51]. Genetic testing is the cornerstone of precision medicine. It has become an increasingly critical component in the diagnosis of NSCLC, particularly in the identification of mutations, which can inform the selection of targeted treatment regimens [52]. Using the SEER database, Kehl et al. [49] presented molecular testing rates of 32.8% among Asian/other descents, 26.2% among White, and 14.1% among Black patients. Analyses also demonstrated median survival times of 8.2 months among patients with molecular testing and 6.1 months among those without testing. Larson et al. [50] utilized data from the Kentucky Cancer Registry and identified risk factors associated with an absence of epidermal growth factor receptor (EGFR) testing, which included male sex, enrollment in Medicaid or Medicare, older age, geographic region, and smoking. Analyses revealed that undergoing EGFR testing was associated with a higher likelihood of overall survival. Results are intriguing considering a study conducted by Cheng et al. [48], which showed that among patients with EGFR-mutated NSCLC, Black patients had shorter survival compared to Non-Black patients (*p* = 0.001), with 2-year survival rates of 33% vs. 61%, respectively. 

Costa et al. [51] conducted a systematic review of the prevalence of targetable mutations among lung cancer patients stratified by race. EGFR was the most common mutation found in Black patients, although less prevalent than rates observed in White, Hispanic, and Asian patients. Black patients were also shown to have a low overall prevalence of anaplastic lymphoma kinase (ALK), c‐ros oncogene 1 (ROS-1), and B-Raf proto-oncogene (BRAF) mutations. Taken together, these results suggest a disproportional eligibility for targeted therapies, the need for more tailored management of lung cancer in the Black patient population, and further support of the benefits of timely molecular testing.

Begnaud [47] examined genetic testing rates among American Indians and Alaska Natives residing in Minnesota, as these groups have been shown to experience higher lung cancer mortality rates than other races. Compared to matched controls, there was no significant difference in mutation testing in American Indians compared to Non-American Indian controls from five tertiary health systems in Minnesota covering a diverse demographic population and geographic area; suggesting that other factors are likely contributing to the higher mortality in this group.

### Lung Cancer Treatment

A total of 17 articles focused on disparities in lung cancer treatment, inclusive of surgical interventions [53–69]. In general, Black patients and patients receiving public insurance appeared to have the worst treatment outcomes, which may be attributable, in part, to lower rates of high-cost treatments and systemic treatments (e.g., chemotherapy, targeted therapy), while Asian patients appeared most likely to receive guideline-concordant treatments (GCT). Using the SEER data, Bradley et al. [56] showed that patients who lived in high-poverty areas were four percentage points less likely to receive high-cost agents, while those not receiving treatment at a National Cancer Institute-designated center were 10 percentage points less likely to receive these agents (*p* < 0.001). This study also indicated a 27 percentage-point increase in the likelihood of receiving a high-cost agent in 2015 compared with 2007. 

Maguire et al. [69] used the California Cancer Registry data to examine use of systemic treatment. More patients receiving systemic treatment had private insurance and fewer had dual Medicare–Medicaid or Medicaid/other public insurance, while more Asian Pacific Islander patients and fewer people in the lowest neighborhood socioeconomic quintile utilized these treatments. Similarly, Verma et al. [68] showed lower use of immunotherapy treatment among Black patients and among patients receiving public insurance. Blom et al. [55] assessed the level of adherence to National Comprehensive Cancer Network guidelines within the NCD, finding that GCT was less likely with increasing age. Additionally, Non-Hispanic Black patients were less likely to receive GCT than Non-Hispanic White patients, and Non-Hispanic Asians were more likely to receive GCT.

Duma et al. [57] conducted a study using the NCD to examine rates of treatment refusal in stage IV NSCLC and showed that a total of 5.4% of patients refused radiotherapy and 10.3% refused chemotherapy, despite trends in survival improvement and provider recommendations. This refusal appeared to be related more to socioeconomic factors than race and ethnicity. Men were less likely to refuse these treatments compared to women. Factors related to refusal of radiotherapy included having Medicaid or Medicare insurance, a low household median income, and lower educational level. Non-Hispanic White, Hispanic, and Asian patients had increasing radiotherapy refusal rates over time, while Non-Hispanic Black patients had less modest increases over time.

Disparities in surgical interventions also were observed. Balekian et al. [54] showed that, in comparison to White men, Black men had 28% lower surgery rates, while White women and Black women underwent surgery at rates comparable to White men. Black men also were less likely than White men to undergo resection following a surgical consultation [58]. They also had higher rates of prolonged intubation and longer hospital stays following non-emergent lobectomy [53]. Numerous factors have been associated with delays in the timing of surgery, including being unmarried, having Medicare or other public insurance, having Medicaid insurance, no insurance, and living in high-poverty areas [63]. In this same study, 28.7% of White patients and 48.4% of Black patients received delayed surgery. Interestingly, Ryan [26] noted in her review that physicians treating Black patients may have reduced access to key clinical resources, in addition to less clinical training. However, Ferguson et al. [60] found that neither physician nor patient race was significantly associated with surgical recommendations or risk estimation of postoperative complications in early-stage lung cancer, an indication that additional explanations for documented racial disparities in lung cancer therapy are warranted. Neighborhood environment may provide some insight, as Whites who live in low Black segregation/high deprivation areas had 15% lower odds of receiving surgery.

Two studies examined disparities in definitive treatment plans for early-stage NSCLC. Using the NCD, Lutfi et al. [62] showed that Black patients were less likely to receive surgery (60.3% vs. 66.9%) and more likely to receive external beam radiation therapy (12.4% vs. 10.6%; *p* < 0.001) compared to their White counterparts, although the surgery rates have been steadily increasing for Black patients over time. Examining the role of site of care, Nguyen et al. [64] found no association between Medicaid expansion under the Affordable Care Act and access to stage-appropriate definitive treatment for various cancers among marginalized racial and ethnic groups cared for at hospitals that predominantly serve Black or Hispanic populations. At the patient level, Medicaid expansion was shown to be associated with improved time to treatment initiation for underserved racial and ethnic groups, but not necessarily at the facility level of hospitals known to predominantly serve them.

### Lung Cancer Survival

There were three articles where the focus was survival [70–72]. In a study by Klugman et al. of NSCLC patients from the Montefiore Medical Center in the Bronx, NY, after adjusting for clinical and social factors, Hispanic/Latino ethnicity was associated with a 30% decreased risk of death compared to Non-Hispanic Whites [70]. Results were not entirely explained by smoking. These same authors published a meta-analysis summarizing the independent contribution of race and ethnicity to survival in U.S. lung cancer patients [71]. After adjusting for smoking status and relevant clinical factors, Asian and Hispanic patients showed improved survival compared to Non-Hispanic White patients. In contrast, no difference in survival was seen between Black and Non-Hispanic White patients [71]. In alignment with this latter study by Klugman [71], Jones et al. [72] demonstrated, in a prospective cohort study, that global African Ancestry was not significantly associated with survival among NSCLC patients across 12 southern states, whereas stage of disease and treatment were significant.

### Hospice Utilization

Johnson et al. [73] performed an exploratory retrospective study that utilized electronic medical records to assess demographic characteristics and hospice use among a geographically distinct population with low hospice enrollment (i.e., 36%). Among those with a verified date of death, more than one-half received care for fewer than seven days. No significant disparities were seen in hospice utilization or length of stay by race, age, or rural/urban areas.

### Smoking and Occupational Risks

Smoking was a primary topic of two articles [66, 74]. Stiles et al. [66] performed a retrospective analysis of demographic and pathological data between smokers and never-smokers. Never-smokers were more likely to be Asian, female, younger, more frequently diagnosed with adenocarcinoma, lower lobe tumors, and have Stage I disease. This analysis indicated an increase in the proportion of never-smokers undergoing resection, and, although demographic differences exist between never-smokers and smokers, these groups had similar survival rates and risk for recurrence and death, following propensity score matching.

Another study examined smoking-related beliefs about lung cancer risk, role of mass media exposure (i.e., reading print media products, listening to the radio and watching television), smoking experience, and health-related discussions with friends and family among patients with low socioeconomic status [74]. Individuals with smoking experience better perceived the lung cancer risks of smoking than those who did not smoke, suggesting that anti-tobacco interventions may require contemporizing to prevent initiation of smoking in non-smokers.

An analysis of the NLST that focused on occupational risks showed that Black patients reported greater exposure, particularly to asbestos and silica and had higher odds of lung cancer diagnosis than White patients [75]. Results also demonstrated that smokers exposed to asbestos and silica were at increased risk for lung cancer.

## Discussion

The findings of this TLR highlight long-standing and persistent health disparities and inequities faced by marginalized populations, particularly Black patients [76–79]. These disparities span the continuum of lung cancer care and are associated with a variety of sociodemographic and socioeconomic factors, which interplay with race and ethnicity. Black patients compared with their Non-Hispanic White counterparts develop lung cancer at an earlier age, are less likely to meet eligibility criteria for lung cancer screening, and, thus, are more likely to present with advanced-stage disease. Furthermore, once diagnosed, they are less likely to receive genetic testing for mutations, surgical intervention, and high-cost and systemic treatments, all of which are associated with greater survival. Of note, a 2022 review of cancer disparities in breast, cervical, ovarian, endometrial, prostate, colorectal, gastrointestinal, and hepatocellular cancers also showed similar disparities among Black patients [80].

The present review, with a focus on the Black community, revealed similar lung cancer disparities in other minoritized racial/ethnic populations. The American Lung Association’s Racial and Ethnicity Disparities 2022 Report documents that Latino Americans, Asian Americans/Pacific Islanders, and American Indians/Alaska Natives with lung cancer are less likely to be diagnosed early and receive any treatment compared to White patients [12]. Latino patients are equally likely as White patients to receive surgical treatment, while Asian American/Pacific Islander patients are more likely and American Indian/Alaska Native patients are less likely to receive surgical treatment compared with White patients. Interestingly, only Asian American patients are more likely than White patients to survive five years post-diagnosis. Moreover, compared to the Non-Hispanic White population, these racial and ethnic groups are at increased risk for lack of health insurance [81]. Although making up 43.1% of the non-elderly U.S. population, they account for more than one-half of the non-elderly uninsured [65]. In 2019, the uninsured rates were lowest for Non-Hispanic White (7.8%) and Non-Hispanic Asian individuals (7.2%) followed by Non-Hispanic Black people (11.4%), Native Hawaiians and Other Pacific Islanders (12.7%), Hispanic individuals (20.0%), and American Indian and Alaska Natives (21.7%) [82].

This review underscores the systemic multifactorial complexities of health disparities, which warrant evidence-based interventions at local, state, and federal levels; sustainable community and health system partnerships; and unconscious bias training for physicians and other healthcare workers [83–88]. While physician-level interventions can mitigate health disparities in clinical practice, medical care accounts for only 10–20% of the overall variability in population health outcomes, with the other 80–90% related to social determinants of health (e.g., health-related behaviors, socioeconomic factors, and physical environment) [86]. Because physicians and healthcare professionals generally are not trained in social work, their interactions are often limited to recommendations for medical treatment and lifestyle changes, even though the effectiveness of these interventions depend largely on personal social assets [83]. To this point, several publications have identified strategies and resources for family medicine practitioners to address social determinants of health in clinical practice [83, 88, 89]. Further evidence is needed, though, to determine if these can serve as a useful framework to develop similar programs for lung cancer specialists and institutions treating underserved and marginalized patients.

### Call to Action

Systemic barriers to timely and appropriate screening, diagnosis, and treatment experienced by Black and other marginalized groups contribute to the observed disparities within the lung cancer population. Recent eligibility revisions to the USPSTF guidelines may decrease some of these disparities in lung cancer screening rates for female, Black, and Hispanic populations and may result in more testing [43]. This literature review highlights the multiple barriers that deserve our attention and warrant collaborative solutions. Professional organizations and task forces, such as the USPSTF among others, should continue to strive to improve screening guidelines to reduce disparities, while healthcare organizations and providers should seek to continually monitor quality metrics in lung cancer screening to ensure optimal uptake. Beyond lung cancer screening, healthcare organizations and providers should be aware that disparities are occurring at each phase of the patient journey, including diagnosis, genetic mutation testing, receipt of treatments including surgical interventions, and survival. Furthermore, resources should be committed to measuring and mitigating these observed disparities to ensure improved patient outcomes. Mitigation of these disparities has the potential to impact quality of life and reduce the clinical and economic burden associated with NSCLC. For instance, patients who fail initial treatment protocols will incur significantly greater costs than those who do not [90], while those presenting with later stage disease may incur up to seven times greater healthcare costs than those diagnosed at earlier stage disease [91]. Productivity losses also have been observed among NSCLC patients and unpaid caregivers, with one investigation demonstrating that one-third of NSCLC patients left the workforce following their diagnosis [92]. Thus, improved screening, diagnosis, and treatment pathways have the potential to directly and indirectly impact various aspects of the patient’s life.

### Strengths and Limitations

There are several advantages to the methodology used for the current review. First, this review utilized a multi-tier approach for identifying articles to include a broader range of article types and sub-topics that would be of interest to various professionals studying lung cancer disparities. Additionally, published articles were limited to those published since 2018, providing a relatively current assessment of the challenges facing the lung cancer population. Finally, this comprehensive review incorporated articles that examined the full spectrum of patient care, from screening to survival, providing insights on lung cancer disparities throughout the patient journey. As new research and initiatives emerge to address and provide solutions to long-standing health inequities at the patient, provider, and population levels, the current synthesis of the recent evidence will offer an opportunity to longitudinally monitor and gauge progress.

However, there are several limitations to note while interpreting the present results. First, as this was a TLR and not a systematic literature review, there may have been some bias present during article selection and extraction, with relevant studies potentially missed. Further, strict selection criteria were used for article selection, which was limited to studies indexed in PubMed®. Also, many cohorts used in the reviewed articles were limited to specific local populations, although this was largely a function of the real-world nature of the studies. In addition, only articles written in English were included. The study selection criteria and article populations should be taken into account when considering the generalizability of this review’s findings. Finally, many studies did not specify lung cancer type, although as approximately 80–85% of lung cancers are NSCLC, it is presumed that the majority of patients in the reviewed studies had a NSCLC diagnosis [93].

## Conclusions

Results of the current TLR present compelling evidence of significant and persistent disparities experienced by marginalized racial and ethnic populations in the U.S. across the entire clinical spectrum of lung cancer. The volume of literature presented underscores an increased desire by physicians and researchers to better understand heterogeneities in clinical care and outcomes among distinct racial and ethnic populations to ultimately improve patient care and survival outcomes. More importantly, the synthesis of evidence in this literature review supports the need for coordinated, multi-level collaboration among private, public, and government entities to track eligibility for and provision of medical care to patients and to measure outcomes across the patient journey; these will facilitate knowledge about gaps in existing services and aid in measuring outcomes that include screening, appropriate genetic testing, treatment options, and overall survival by site of care. The growing awareness and understanding of health equity provide optimism for addressing and reducing disparities in lung cancer between Black and other marginalized groups and their White counterparts. Thus, this review with current evidence serves as a call to action to identify effective evidence-based strategies to guide our efforts in reducing disparities among U.S. populations that are affected by lung cancer. 

### Supplementary Information

Below is the link to the electronic supplementary material.

Supplementary file1 (DOCX 57 KB)
