# Supplementary material for: Disparities in Lung Cancer: A Targeted Literature Review Examining Lung Cancer Screening, Diagnosis, Treatment, and Survival Outcomes in the United States
Source: J Racial Ethn Health Disparities. 2023 May 19;11(3):1489–500. doi: 10.1007/s40615-023-01625-2 (PMC11101514; doi:10.1007/s40615-023-01625-2)
Supplement: Supplementary file 1 — Supplementary file1 (DOCX 57 KB) [file 40615_2023_1625_MOESM1_ESM.docx]

Health Disparities in Lung Cancer: A Narrative Review Examining Lung Cancer Screening, Diagnosis, Treatment, and Survival Outcomes in the United States

Journal of Racial and Ethnic Health Disparities

Lisa Dwyer, MPH; Pratyusha Vadagam, MS, Julie Vanderpoel, PharmD, MPA; Janssen Scientific Affairs, LLC, Titusville, NJ
Carol Cohen, BA; Benjamin Lewing, PhD; Joseph Tkacz, MS; Inovalon, Bowie, MD

CORRESPONDING AUTHOR:
Lisa L. Dwyer, MPH, HEOR-C
Director, Population Health Research
Real World Value & Evidence
Janssen Scientific Affairs, LLC
1125 Trenton Harbourton Road
Titusville, NJ 08560
Mobile: (561) 290-9901
E-mail: [LDwyer2@its.jnj.com](mailto:LDwyer2@its.jnj.com)

Supplemental Table 1. Key elements of the 49 articles chosen for review. Note that racial and ethnic groups utilize the nomenclature reported from each article.

| STUDY FOCUS | AUTHOR  (YEAR) | DATABASE SOURCE | STUDY  DESIGN | RACE/SOCIAL DETERMINANTS | PATIENT POPULATION | STUDY  PERIOD | OUTCOMES |
| --- | --- | --- | --- | --- | --- | --- | --- |
| Demographics  Lung Cancer Screening | Haddad DN et al^15^ (2020) | Review  (Literature Search) | Retrospective | Racial/ethnic minorities, rurality | Age not specified  White  Black  Hispanic  American Indian/Alaskan Native (AI/AN)  Asian/Pacific Islander (API)  High risk populations (human immunodeficiency virus [HIV]) | 2003-2019 | • Black males have the highest rates of age-adjusted lung cancer incidence for smokers and never-smokers and lung cancer mortality among all US racial/ethnic groups.  • Blacks develop lung cancer at an earlier age than Whites (median age, 67 vs. 70 yrs.); likely to present with advanced-stage disease (53% among Blacks vs. 49% Whites).  • Hispanics and AI/AN are underrepresented in published literature.  • Hispanics have a lower smoking prevalence and lung cancer mortality than Blacks and Whites.  • AI/AN have the highest overall prevalence of cigarette smoking yet have lower incidence and mortality rates than Blacks and Whites.  • HIV-positive patients are also at high risk and experience lung cancer incidence three times higher than the general population.  • Disparities in lung cancer screening associated with Race/Ethnicity; Rurality; Environmental (i.e., radon) and Occupational exposure (i.e., asbestos); HIV Infection; Access to Care; Patient-Level Barriers (socioeconomic status; geographic location, insurance).  • Screening eligibility guidelines should consider racial differences in smoking behaviors, lung cancer incidence, age at presentation, and mortality. |
| Lung Cancer Incidence | Ryan BM^16^  (2018) | Review  (Literature Search) | Retrospective | Race/Ethnicity | European Americans (EA)  Afro Americans (AA) | Articles published between  1985-2018 | • Racial/ethnic disparities exist across many U.S. populations in men (Hispanics [lowest incidence], Asian/Pacific Islander, American Indian/Alaska Native, Non-Hispanic Whites, Non-Hispanic Blacks [highest]) and women. (Hispanics [lowest incidence], Asian/Pacific Islander, Non-Hispanic Blacks, American Indian/Alaska Native and Non-Hispanic Whites [highest]).  • Etiological factors contributing to disparities in lung cancer incidence are smoking (screening age, dose, duration, cessation, menthol, genetics and metabolism); Environment (body mass index, alcohol consumption, radon, pollution, and geographic location); Early detection (screening eligibility, screening uptake, and biomarkers); Societal factors (stage at presentation, insurance status, belief systems, socioeconomic status, access to care, use of care, and health literacy); Biology (genetics and metabolism, transcriptomics, and biomarkers).  • AA start smoking later in life; smoke fewer and menthol cigarettes per day; have higher total nicotine equivalent levels than EA.  • AA are 3 times as likely to live in poverty as EA.  • AA live in rural counties often located near or in poor working-class communities and disadvantaged groups and at increased risk for environmental hazards.  • Physicians treating AA patients may have less clinical training and access to key clinical resources.  • Limited Precision Medicine studies in minority populations.  • AAs are more likely to present with advanced disease than EAs.  • AAs have the highest lung mortality and worst survival compared to other racial/ethnic groups; however, when access to care is controlled such as within the US military healthcare system, survival times are more equitable. |
| Lung Cancer Screening | Sosa E et al^17^  (2021) | Systematic Review (Literature Search) | Retrospective | Race, socioeconomic status (SES) | Age 45-80  Current and former smokers | January 1, 2010-Feb 27, 2020 | • 3721 studies screened; 21 eligible studies reviewed.  Eligibility  • Seven studies (33%) investigated eligibility for screening according to the 2013 US Preventive Services Task Force (USPSTF) Lung Cancer Screening (LCS) guidelines.  Utilization, perception, and utility  • Five studies (24%) evaluated race and/or SES impact on LCS utilization.  • Two studies (10%) examined patient beliefs about LCS.  • Five studies (24%) investigated impact of race and/or SES on stage of Non-Small Cell Lung Carcinoma (NSCLC).  Post screening behavior and care  • Three studies (14%) examined smoking behavior and cessation differences by race/ethnicity.  • Three studies (14%) reported on completion of follow-up care.  • One study evaluated impact of race on surgical treatment in patients who had NSCLC.  • Two studies (10%) investigated impact of race on lung cancer mortality among individuals at high risk for lung cancer.  • Disparities are prevalent in LCS eligibility; utilization, perception, and utility; and post screening behavior and care.  • Disparities collectively cause significant NSCLC survival gaps along racial and socioeconomic lines. |
| Lung Cancer Risk | Sanderson M et al^20^ (2018) | Southern Community Cohort Study (SCCS) linked to 12 southeastern state cancer registries (Alabama, Arkansas, Florida, Georgia, Kentucky, Louisiana, Mississippi, North Carolina, South Carolina, Tennessee, Virginia, and West Virginia) and National Death Index | Retrospective | Neighborhood deprivation, race, sex | Age 40-79  White men  White women  Black men  Other race men  Other race women | 2002-2009 | • A disparity in cancer risk by neighborhood deprivation was detected among current and former smokers (Odds Ratio [OR]: 1.29), comparing 4th quartile of deprivation to 1st quartile, and OR: 1.36 comparing 3rd quartile to 1st quartile (Trend p= 0.04).  • Neighborhood deprivation was not associated with increased lung cancer risk by sex or race. |
| Lung Cancer Incidence | Houston KA et al^21^  (2018) | Centers for Disease Control and Prevention, National Programs of Cancer Registries (NPCR), and National Cancer Institute’s Surveillance, Epidemiology, and End Results (SEER) registries | Retrospective | Race/Ethnicity, urban-rural,  gender | Age ≥18  Non-Hispanic (NH) white  NH black  Hispanic | 2004-2013 | • Among women whose lung cancer was diagnosed <55 years of age, incidence of squamous cell carcinoma and adenocarcinoma was higher for NH blacks compared to NH whites (Rate Ratio [RR]; squamous cell 1.19; adenocarcinoma 1.10; large cell carcinoma (RR 1.15).  • Incidence for Small Cell Lung Carcinoma (SCLC) was significantly lower for male Hispanics (RR 0.45) and NH Blacks (RR 0.80) than for NH Whites.  • Rates of squamous cell among men were significantly higher in adjacent metropolitan (RR 1.25) and nonadjacent (RR 1.19) counties than the rates living in metropolitan counties; Similar respective trend for women (1.11 and 1.09).  • Degree of disparity among NH blacks versus NH whites increased with rurality of residence, rising from 24% to 29% to 45% in metropolitan, adjacent metropolitan, and nonadjacent metropolitan locations, respectively.  • Annual percent change (APC) rates of adenocarcinoma increased fastest in counties nonadjacent to metropolitan areas. APC rate for rural area was 1.9 for male and 3.7 for female, compared to 1.0 and 2.4 for metropolitan area, respectively. |
| Lung Cancer Incidence | Yoon HS et al^22^ (2019) | Southern Community Cohort Study (SCCS) linked to 12 southeastern state cancer registries (Alabama, Arkansas, Florida, Georgia, Kentucky, Louisiana, Mississippi, North Carolina, South Carolina, Tennessee, Virginia, and West Virginia) and National Death Index | Retrospective | Socioeconomic status, race | Age 40 - 79  Race  African American European American  Other  Household income  < $15,000  $15,000–$25,000  > $25,000 | March 2002-September 2009 | • After adjusting for covariates, tooth loss, tooth decay, and history of periodontal disease were significantly associated with an increased risk of lung cancer among the SCCS population.  • >10 teeth lost Odds Ratio (OR) 1.64 (95 % Confidence Interval (CI): 1.00–2.69); ≥ 6 decaying teeth OR of 1.65 (CI: 1.18–2.31).  • Findings more evident among low-income population of African Americans (OR=1.56, 95% CI: 1.05, 2.31) and heavy smokers (OR=2.05, 95% CI: 1.38–3.05). |
| Lung Cancer Screening | Annangi S et al^23^  (2019) | Surveillance, Epidemiology, and End Results (SEER) | Retrospective | Race | Age 40 - 85+  White  Black | 2004-2014 | • Total of 9% of cancers were diagnosed in the age range of 45-54 (early-onset).  • Early-onset lung cancers (diagnosed between ages 45 and 54) significantly more frequent among African Americans compared to Whites (African American vs. White, 4.7% vs. 2.7%, p<0.05 age group 45-49; 9.5 vs.5.5%, p<0.05 age group 50-54).  • African Americans have significantly higher age-specific incidence rates compared to Whites until convergence at age group 70–74.  • African Americans more likely to be diagnosed at advanced lung cancer stage III (Whites vs. African Americans 23.4% vs. 25.9%; p<.05) and stage IV (Whites vs. African Americans 42.9% vs. 46.0%; p<.05). |
| Lung Cancer Screening | Borondy Kitts AK^24^  (2019) | Review (Literature Search) | Retrospective | Race | Lung Cancer | Articles published between 2003-2018 | • Recommendation by and trust in physician and enthusiasm of the recommendation influence the decision to screen. • Lack of awareness of screening option by patient and primary care physician community. • African Americans (AA) less likely to meet screening criteria due to younger age and lower tobacco exposure • Barriers to shared decision making reported for both patients and clinicians • Smokers, and especially former smokers, underestimate their perceived risk of lung cancer and overestimate the curability.  • AAs are diagnosed at a later stage, and there is a greater likelihood they will refuse treatment options when diagnosed. • Disadvantaged populations are at higher risk for lung cancer mortality facing stigma associated with smoking, race, disability, or socioeconomic status. • Codeveloping interventions with local and state organizations to raise awareness and develop outreach programs and educational materials are recommended to avoid increasing lung cancer mortality disparity in the AA and other disadvantaged communities. • Challenges to screening include access to care, awareness of the option for screening, stigma and implicit bias that are due to stigmatization of smoking, stigma of race, nihilism with lung cancer diagnosis viewed as a “death sentence,” shared decision making, and underestimation of lung cancer risk. • AAs are diagnosed at a later stage, and there is a greater likelihood they will refuse treatment options when diagnosed. • Fewer AAs were found to meet lung cancer screening eligibility criteria compared with whites because of lower tobacco exposure and younger age at time of diagnosis. • Disadvantaged populations are at higher risk for lung cancer mortality. They also face both the stigma associated with smoking and the stigma associated with their race, disability, or socioeconomic status. |
| Lung Cancer Screening | Guichet PL et al^25^ (2018) | Centinela Valley (South Los Angeles County) Lung Cancer Screening Program,  providing free lung cancer screening to residents from underserved communities in south Los Angeles County | Prospective | African American, low-income, underinsured population, underserved | Age, mean 59 (50-78; 52% male)  At-risk patients meeting National Comprehensive Cancer Network (NCCN) eligibility criteria for  lung cancer screening | July 21, 2015-April 3, 2017 | • Lung cancer screening profile differed from that observed in the National Lung Screening Trial (NLST) in more privileged communities.  • Racial-ethnic makeup of screening program (84% black, 11% Hispanic/Latino, and 5% white) differed from NLST patient population (5% black, 2% Hispanic/Latino, and 91% white).  • Malignancies detected in minority, socioeconomically disadvantaged communities were advanced lung cancers (one stage IIIB small cell lung cancer and one stage IV lung cancer of unknown type) in contrast to NSLT that predominately catches early-stage lung cancer suggesting that NLST is not generalizable to US population at large. |
| Lung Cancer Screening | Japuntich SJ, et al^26^ (2018) | Hospital electronic records from Lifespan Medical System | Retrospective Survey | Race | Age 55 - 80  Black  Non-Black | 2016 | • Among eligible patients, 35% met Lung Cancer Screening (LCS) criteria.  • Non-Blacks were 90% more likely to meet criteria for LCS than Blacks (44% Non-Blacks vs. 27% Blacks).  • Of patients meeting United States Preventative Services Task Force criteria, 21% reported being screened (Non-Blacks 25.0%, Blacks 16.7%; adjusted Odds Ratio=1.6, 95% Confidence Interval 0.7, 3.9).  • Eligible Non-Blacks were 2.8 times more likely to be screened than eligible Blacks (30% vs. 12%). |
| Lung Cancer Screening | Lake M et al^27^ (2020) | Clinical databases (chart review) Jane and Leonard Korman Respiratory Institute Lung Cancer Screening Program (LCSP); Thomas Jefferson University; Philadelphia, PA | Retrospective | Race | Age Mean 64.3 ± 5.9  Blacks and Whites referred for screening  Blacks and Whites undergoing screening  Cohorts based on RAD score Lung-RADS 1  Lung-RADS 2  Lung-RADS 3  Lung-RADS 4A, 4B, and 4X | Referrals between May 2015-July 2017  Chart review for events that occurred through September 6th, 2019. | • Among Black patients referred for screening, 36.2% were not screened compared to 23.3% of White patients.  • Black patients who underwent a screening Low-Dose Computerized Tomography demonstrated longer follow-up time intervals compared with White patients across all **Lung Imaging Reporting and Data System** (Lung-RADS) categories.  • Black patients with Lung-RADS 4 had lower rates of returning to annual screening and higher rates of loss to follow-up compared with white patients. |
| Lung Cancer Screening | Morgan RL et al^28^ (2020) | Surveillance, Epidemiology, and End Results (SEER) linked Medicare database | Retrospective | Race/Ethnicity | Age ≥66 at diagnosis  Non-Hispanic White  Black  Hispanic | 2007-2015 | • Approximately 78% of non-Hispanic Whites received a positron emission tomography (PET) at diagnosis compared with 63% of Blacks and 70% of Hispanics.  • Adjusted for demographic, socioeconomic, and facility characteristics, black patients with squamous cell Non-Small Cell Lung Carcinoma (NSCLC) were about one-half as likely to receive a PET and, Hispanic patients were about two-thirds as likely to receive a PET compared with non-Hispanic whites.  • Patients with non-squamous cell NSCLC, the likelihood of receiving a PET was also lower for Black and Hispanic patients.  • Differences in PET use were noted throughout all stages for squamous cell carcinoma patients  and other histology types.  • Combining all histology types, probability of survival was more than 20% higher in patients imaged with PET versus computerized technology (CT) alone.  • Patients receiving treatment at a National Cancer Institute designated center were 10% more likely to undergo PET imaging than teaching or community hospitals. |
| Lung Cancer Screening | Pasquinelli MM et al^29^ (2018) | Clinical trial data National Lung Screening Trial (NLST) and federally qualified health centers at University of Illinois at Chicago (UIC) | Retrospective | Race/Ethnicity | Age UIC Mean 62.8;  NLST Mean 61.4  African American Asian | September 4, 2015 to December 28, 2017 | • UIC had different racial and ethnic composition than the NLST low-dose computerized tomography (LDCT).  • African American (UIC, 69.6% [348 of 500] vs NLST, 4.5% [1195/ of 26 722]) and Hispanic or Latino (UIC, 10.6% [53 of 500] vs NLST, 1.8% [479 of 26 722])  • UIC cohort had a higher lung cancer detection rate (2.6% [13 of 500]) than the NLST low-dose computerized tomography (LDCT) (1.1% [292 of 26 455])  • Proportion of positive (Lung-RADS class 3 or 4) LDCT screens in the UIC cohort (24.6% [123 of 500]) was nearly double that in the NLST LDCT arm (13.7% [3601 of 26 455]).  • Both cohorts had greater than 50% of lung cancer cases detected at an early (stage I) curable stage (UIC [7 of 13] and NLST [155 of 266]). |
| Lung Cancer Screening | Pasquinelli MM et al^30^ (2020) | Electronic medical records  with review of clinical notes from University of Illinois Hospital and Health Sciences System | Retrospective | Race/Ethnicity | Age <45 to >80  African American Non-Hispanic Mean 64.3 White Non-Hispanic Mean 64.0 Other Mean 68.2  Ever smokers African American, Non-Hispanic, White Non-Hispanic Other (Includes Hispanic, Asian, Unknown | 2010-2019 | • Sensitivity to detect lung cancers overall is lower for United States Preventative Services Task Force (USPSTF) criteria than for the Prostate, Lung, Colorectal and Ovarian (PLCOm2012 model) at all three risk thresholds.  All  USPSTF sensitivity = 52.3  PLCOm2012 sensitivity = 69.3  White  USPSTF sensitivity = 62.4  PLCOm2012 sensitivity = 70.5  African American  USPSTF sensitivity = 50.3  PLCOm2012 sensitivity = 74.1  • PLCOm2012 model was found to be preferable over the USPSTF criteria at identifying African American ever-smokers for lung cancer screening. |
| Lung Cancer Screening | Pinsky PF et al^31^ (2021) | Adult and cancer control supplement files of the 2015 National Health Interview Survey (NHIS) | Retrospective | Race/Ethnicity | Age 50-80  Sample size based on collective Guidelines of Centers for Medicare and Medicaid Services (CMS), National Comprehensive Cancer Network (NCCN), and US Preventive Services Task Force (USPSTF) current or with expansion of age and smoking or quit thresholds as well as PLCOM2012 risk model for Lung Cancer Screening (LCS) | 2015 | • Population estimates for LCS eligibility per guideline per million USPSTF (2013) N=8.3; USPSTF (2020) N=14.8  USPSTF 50/20/15 (Expansion proposed in 2020 to include lower age of 50 years and a 20-pack-year smoking history threshold while maintaining the 15-years-since quitting threshold in former smokers) 05/20/2025) N=15.5  NCCN N=13.3  PLCOMM2012 (Prostate, Lung, Colorectal and Ovarian) N=12.8  • Overall eligibility rates on average about 10 percentage points higher for men than women.  • For both men and women, and both overall and among ever smokers, non-Hispanic Whites had the highest eligibility rates across all guidelines followed generally by non-Hispanic Blacks, then Asians and Hispanics.  • Among both men and women, non- Hispanic Whites had the highest eligibility screening rate to lung cancer incidence rate ratios across all guidelines.  • Non-Hispanic Black men had higher lung cancer incidence than non-Hispanic White men. |
| Lung Cancer Screening | Poghosyan H et al^32^ (2021) | Behavioral Risk Factor Surveillance System (BRFSS), Lung Cancer Screening Module Sponsored by the Centers for Disease Control and Prevention and state health departments (Florida, Georgia, Maryland, Missouri, Nevada, Oklahoma, Vermont and Wyoming) | Retrospective | Race/Ethnicity | Age 55-74  Current smokers who reported having smoked at least 100 cigarettes in their entire life and at the time of survey reported smoking cigarettes every day or some days.  Whites  Blacks  Other | September 4, 2015 to December 28, 2017 | • Median response rates of survey for the 8 states ranged from 38.2% to 64.1%.  • Compared to Blacks, Whites appear more likely to meet Low-Dose Computerized Tomography (LDCT) screening eligibility criteria and appear more likely to receive an LDCT scan.  • After adjusted analysis, Blacks had about half the odds (0.52) of receiving LDCT scan in the past 12 months compared with Whites.  • Odds of receiving LDCT scan were higher for those who were male, who tried to quit smoking in the past year, and for those with more education, health insurance, high blood pressure, lung disease, or cancer history (other than skin or lung cancer) |
| Lung Cancer Screening | Reese TJ et al^33^ (2021) | Centers for Disease Control and Prevention’s Behavioral Risk Factor Surveillance System (BRFSS) from 19 participating states with optional lung cancer screening module for 2017 (Florida, Georgia, Kansas, Kentucky, Maine, Maryland, Missouri, Nevada, Oklahoma, Vermont, and Wyoming) and 2018 (Delaware, Maine, Maryland, New Jersey, Oklahoma, South Dakota, Texas, and West Virginia) | Retrospective | Race/Ethnicity Sex | Age 50-80 with smoking history   Non-Hispanic White  Non-Hispanic Black  Hispanic | January 1, 2017, to December 31, 2018 | • Revised criteria increased eligibility by sex and race/ethnicity.  Men (from 29.4% to 38.3% [8.9% difference]; p< .001)  Women (25.9% to 36.4% [10.5% difference]; p< .001)  Whites (31.1% to 40.9% [9.8% difference]; p< .001)  Blacks (16.3% to 28.8% [12.5% difference]; p< .001)  Hispanics (10.5% to 18.7% [8.2% difference]; p< .001)  • Adjusted odds ratios for screening eligibility of women (0.88; 95% Confidence Interval (CI), 0.79-0.99; P=0 .04) lower than men and for both Blacks (0.43; 95% CI, 0.33-0.56; P<0 .001) and Hispanics lower than whites (0.70; 95% CI, 0.62-0.80; P<0 .001). |
| Lung Cancer Screening | Riviera MP et al^34^ (2020) | Position Statement  (Literature Review) | Retrospective | Race/Ethnicity, socioeconomic, sex-based differences in smoking behavior and lung cancer risk | Lung Cancer | Study conducted in 2019- 2020; 163 articles published between 1995 to 2020 | • Existing lung cancer screening (LCS) guidelines do not consider racial, ethnic, socioeconomic, and sex-based differences in smoking behaviors (including intensity and years since quitting) or lung cancer risk in special populations (HIV infection).  • Multiple barriers, including access to screening and cost, further contribute to the inequities in implementation and dissemination of LCS.  • Socially and economically disadvantaged populations are among the most vulnerable populations at risk for poor lung cancer outcomes.  • Significant disparities across the continuum of LCS implementation—not getting screened for tobacco use, not meeting eligibility criteria, not having access to quality screening and tobacco treatment, and lack of insurance, among many—threaten to worsen disparities in lung cancer.  • American Thoracic Society Statement proposed strategies to reduce LCS Disparities (https://www.atsjournals.org/doi/pdf/10.1164/rccm.202008-3053ST) |
| Lung Cancer Screening | Rohatgi KW et al^35^ (2020) | Location/Geographic data; Census block group and county-level data from Missouri and Illinois  Matched zip codes by addresses of screening  centers accredited by the American College of Radiology and Lung Cancer Alliance (now GO2 Foundation for Lung Cancer)  Screening Centers of Excellence | Retrospective | Urban-Rural | Age 55-79  Counties in Missouri and Illinois  Large central metro  Large fringe metro  Medium metro  Small metro  Micropolitan  Noncore | 2013-2019 | • Approximately 97.6% of metropolitan residents had access to screening, compared with 41.0% of nonmetropolitan residents.  • After controlling for sociodemographic characteristics, the odds of having access to screening in rural areas were 17% of the odds in metropolitan areas.  • There was no association between screening access and lung cancer mortality. |
| Lung Cancer Screening | Warner ET et al^36^ (2019) | Health Information National Trends Survey (HINTS) a nationally representative cross-sectional survey conducted by the National Cancer Institute | Retrospective | Race/Ethnicity | Age 55-80  Hispanic  Non-Hispanic White  Non-Hispanic Black  Non-Hispanic Asian  Other | Three study periods 2013, 2014, 2017 | • Most current and former smokers have not discussed lung cancer screening with a healthcare provider, and there have not been increases over time as would be expected with increasing receipt of lung cancer screening. • Overall proportion of current and former smokers that reported discussing lung cancer screening with a health provider was 17.8% in the 2017 survey cycle and had not increased since 2013. • Females were 32% less likely to report a lung cancer screening discussion and the association was strongest among Non-Hispanic White females. |
| Genetic Testing | Begnaud A et al^37^ (2020) | Electronic Health Records | Retrospective | Race | Age >18  American Indians and Alaska Natives (AI/AN)  Non-AI/AN | < 2010-2013+ | • Overall testing rates: 35% in cases; 22% in controls.  • No significant difference in mutation testing in AI cases compared to non-AI controls.  • Most cases and controls current or former cigarette smokers. |
| Genetic Testing | Cheng H et al^38^ (2020) | New York-Bronx (Montefiore) Network Cancer Registry | Retrospective | Race, socioeconomic status (annual household income based on home zip code) | Mean Age  Blacks with EGFR mutations 63.2; Non-Blacks 68.2 | 2009-2015 | Epidermal growth factor receptor (EGFR) Mutational rate  • Overall 15% (98/652).  • Blacks 14% (35/258).  • Non-Blacks 16% (63/394).  Survival Rate  • 2-yr survival rate among EGFR wild type was similar by race (Black 38.2% [52/136] vs. Non-Black 39% [75/192]).  • 2-yr survival rate among EGFR mutations was shorter by race (Black 33.3% [9/27)] vs. Non-Black 61.3% [27/44]).  • Similar survival findings following adjusted analysis. |
| Genetic Testing | Kehl KL et al^39^ (2019) | Surveillance, Epidemiology, and End Results Program (SEER) registry linked to Medicare claims | Retrospective | Race, Poverty | Age 66-99  White  Black  Asian/other  Non-Hispanic  Hispanic | 2008-2013 | • 1437 (25.9%) individuals had molecular testing.  • Testing rates were 14.1% among Blacks; 26.2% among Whites; and 32.8% among Asian/other descent.  • Testing rate was 28.4% for Medicaid-ineligible and 20.6% for Medicaid eligible patients.  • Testing rates were 19.9% among patients in the highest census tract-level poverty rate quintile vs. 30.7% among patients in the lowest quintile.  • Median survival for patients with early testing was 8.2 months vs. 6.1 months for those without.  • Testing rates increased over the study period.  • Association of testing with survival depended on treatment; patients who received both genomic testing and  tyrosine kinase inhibitors lived longer (18.8 months) than patients who received genomic testing and chemotherapy (9.8 months) or genomic testing without systemic therapy (5.2 months). |
| Genetic Testing | Larson KL et al^40^ (2020) | Kentucky Cancer Registry (KCR); part of Centers for Disease Control and Prevention National Program of Cancer Registries and National Cancer Institute Surveillance and Epidemiology and End Results [SEER] Program)  KCR linked to claims  from Medicaid, state employee insurance, private insurance groups as well as SEER Medicare database. | Retrospective | Rurality (Appalachian region carries highest cancer burden in Kentucky), poverty, education, type of insurance | Age 20-75+ | Cases diagnosed 2007-2011 | • Testing for epidermal growth factor receptor (EGFR) mutations increased from 0.1% to 10.6% over study period.  • Erlotinib use ranged from 3.4% to 5.4% with no trend over time.  • Not being white or black associated with EGFR testing and erlotinib prescribing.  • High poverty, low high school attainment, and Medicare or Medicaid insurance were significantly less likely to have EGFR testing or an erlotinib prescription.  • Living in rural areas regardless of Appalachian status less likely to receive EGFR testing.  • Patients receiving EGFR testing had better survival.  • Better survival with private insurance and living in non-Appalachian metropolitan area. |
| Genetic Testing | Costa PA et al^41^ (2021) | Systematic Review and Meta-Analysis (Literature Search PubMed/MEDLINE, Cochrane Library, EMBASE, CENTRAL, Google Scholar, and clinicaltrials.gov databases) | Retrospective | Race | Age not specified  Black  White  Hispanic  Asian | Up to November 19, 2018, with no lower date truncation | • Prevalence epidermal growth factor receptor (EGFR) mutation was 6% (95% Confidence Interval (CI), 5 to 7) in Black,12% (95% CI, 11 to 13) in White, 35% (95% CI, 33 to 37) in Hispanic, and 46% (95% CI, 40 to 51) in Asian patients.  • Prevalence BRAF mutation was 1% (95% CI, 0 to 2) in Black, 3% (95% CI, 2 to 3) in White, 4% (95% CI, 1 to  18) in Hispanic, and 2% (95% CI, 0 to 11) in Asian patients.  • Prevalence ROS-1 mutation was found in 0% (95% CI, 0 to 1) of Black,1% (95% CI, 0 to 3) of White, not reported in Hispanic, and not found in any Asian patients.  • Prevalence anaplastic lymphoma kinase (ALK) was 1% (95% CI, 0 to 2) in Black, 2% (95% CI, 1 to 3) in White, 7% (95% CI, 2 to 23) in Hispanic, and 6% (95% CI, 1 to 11) in Asian patients.  • EGFR mutation prevalence lower in Black patients compared with Whites, Hispanics, and Asian patients (p<.01).  • BRAF mutations less prevalent in Black compared with White patients (p<.05).  • ALK mutations less prevalent in Black compared with Hispanic patients (p<.05). |
| Treatment | Alwatari Y et al^43^ (2021) | American College of Surgeon National Surgical Quality Improvement Program (ACS-NSQIP) database  Centers for Disease Control and Prevention (CDC) Surveillance, Epidemiology, and End Results (SEER) Program | Retrospective | Race/Ethnicity | Mean Age 67.6 ± 9.7  White  Black  Hispanic  Asian | 2005-2016 | • After adjusting for covariates, Blacks had higher rates of prolonged intubation compared to Hispanics (Odds Ratio [OR] (Confidence Interval [CI]), 2.4 (1 - 5.9), p= 0.05) and longer hospital stay compared to Whites (Length of stay > 6; 1.3 (1 - 1.7), p= 0.04).  • Whites had a higher rate of pneumonia compared to Hispanic/Asian (OR (CI)): 2.37 (1.3-4.3).  • Proportion of Blacks and Hispanics in the American College of Surgeon National Surgical Quality Improvement Program was lower than their respective proportion of resectable lung cancer in the United States. |
| Treatment | Balekian AA et al^44^ (2019) | National Lung Screening Trial (NLST) | Retrospective | Race, Sex | Age 55-74 with at least 30 pack-years tobacco use  Clinical stage I (A or B) White men  White women  Black men  Black women  Surgical resection  White men  White women  Black men  Black women | Data collection period not specified | • Surgical resection occurred less frequently in Black men (65%), followed by Black women (90%), White women (93%), and White men (93%). • After adjustment for covariates and enrollment center, the odds of surgery were lower for patients who were ≥65 years of age (Odds Ratio [OR], 0.41; 95% Confidence Intervals [C]I, 0.20-0.83) or current smokers (OR, 0.52; 95% CI, 0.28-0.98). • Compared to White men, Black men had significantly lower probability of receiving surgery (adjusted OR: 0.13; 95% CI, 0.04-0.47). • After adjusting for covariates, the relative risk of undergoing surgery for Black men compared with White men was 0.72 (95% CI, 0.50-0.99). • When examining two-way interactions between sex and race, Black men underwent surgery less often than White men (OR, 0.15; 95% CI, 0.05-0.43), but Black women (OR, 0.85; 95% CI, 0.17-4.37) and White women (OR, 0.83; 95% CI, 0.41-1.68) underwent resection at similar rates. • Random effects model showed a significant intercept term (p< .001), suggesting center effect on undergoing surgery. |
| Treatment | Blom EF et al^45^ (2020) | U.S. National Cancer Database | Retrospective | Race/ethnicity, age, health insurance | Age <50 to ≥80  Non-Hispanic White  Non-Hispanic Black  Non-Hispanic Asian  Hispanic  Other  Unknown | 2010-2014 | • Guideline-concordant treatment was less likely with increasing age and was present after adjusting for covariates - present in all clinical sub-groups.  • Non-Hispanic Black patients were less likely to receive guideline-concordant treatment than Non-Hispanic White patients and was present after adjusting for covariates - disparity was present in all clinical subgroups, although statistically nonsignificant for extensive disease small-cell lung cancer.  • Non-Hispanic Asians were more likely to receive guideline-concordant treatment after adjusting for covariates.  • Non-Hispanic Asian and Hispanic patient treatment varied within clinical subgroups. |
| Treatment | Bradley CJ et al^46^ (2020) | Surveillance, Epidemiology, and End-Results (SEER) registry linked to Medicare  claims data | Retrospective | Race/ethnicity, urban or rural residency, census tract poverty, treatment facility type (e.g., National Cancer Institute [NCI] designation) | Age ≥66  Monthly drug cost  <$5000 vs ≥$5000  White non-Hispanic  Black non-Hispanic  Hispanic  Other/unknown  Treated at an NCI-designated center | 2007-2015 | • Women, White Non-Hispanics, those residing in urban areas and counties with <20% of the population living below the poverty level, and those with fewer comorbid conditions more likely to receive high-cost treatments.  • Treatment at an NCI-designated center or residing in a county with a medical school-affiliated hospital had higher probability of receiving high-cost antineoplastic agent.  • Medicare spent $3500 more on patients who received high-cost agents for 12 months post discharge.  • Use of high-cost agents increased over time with a 27-point likelihood increase from 2007 to 2015 highlighting change in practice patterns.  • Patients who lived in areas of high poverty were 4 percentage points less likely to receive high-cost agents.  • Patients who were not treated at a NCI-designated center were 10 percentage points less likely to receive these agents. |
| Treatment | Duma N et al^47^ (2020) | National Cancer Data Base (NCDB), clinical oncology database jointly sponsored by American College of Surgeons and the American Cancer Society sourced from hospital registry data that are collected in more than 1500 Commission on Cancer-accredited facilities | Retrospective | Socioeconomic status, Race/Ethnicity, Sex | Age median range 64-69  Non-Hispanic White  Non-Hispanic Black  Hispanic  Asian  Other  Radiotherapy Refusal  Chemotherapy Refusal | 2004-2014 | • 5.4% radiotherapy refusal and 10.3% chemotherapy refusal despite provider recommendations.  • Proportion of patients refusing radiotherapy and chemotherapy increased over study period from 4.2% to 7.3% and 7.9% to 15%, respectively (p< .001).  • After adjusting for covariates, men were less likely to refuse treatment compared to women (Odds Ratio = 0.80; 95% Confidence Interval, 0.76-0.84; p<.001).  • Medicaid or Medicare as primary insurance, uninsured status, low household median income, and lower educational level significantly associated with radiotherapy refusal.  • Non-Hispanic Whites, Hispanics, and Asians had increasing radiotherapy refusal rates over time.  • Non-Hispanic Blacks had less pronounced trends over time ranging from about 4% to about 5.9% for radiotherapy.  • Uninsured, Medicaid patients, and patients with high comorbidity index were more likely to refuse chemotherapy.  • Asians had lower rates of chemotherapy refusal relative to Non-Hispanic Whites.  • Non-Hispanic Whites, Hispanics, and Asians had increasing chemotherapy refusal rates over time, while Non-Hispanic Blacks had less pronounced trends over time. |
| Treatment | Ezer N et al^48^ (2020) | Surveillance, Epidemiology, and End Results (SEER)-Medicare linked registry for cancer information from 17 regional registries  American Medical Association (AMA) Physician Masterfile and linked to Medicare files using the Unique Physician Identification Number for surgeon information | Retrospective | Race/Ethnicity | Age >65  White  Black  Surgery within 6-months of diagnosis vs.  No surgery within 6-months of diagnosis | 1998-2009 | • Rates of resection were 80% White vs. 66% Black (p<0.001). • Surgeon age, sex, type of practice, location of training, and years in practice were not different among surgeons who evaluated Blacks vs. Whites (all, p>0.05). • Blacks were less likely to be seen by thoracic surgeons (p<0.05). • After surgical consultation, Blacks were less likely to undergo resection (adjusted Odds Ratio, 0.57; 95% Confidence Interval 0.47–0.69). • There was a significant interaction between individual surgeons and race (Black vs. White) (p<0.05).  • Resection rates varied significantly between surgeons (p<0.001). • Significant interaction between the surgeon intercept and race (p<0.05) showed variability beyond chance across surgeons in resection rates of Black vs. White. |
| Treatment | Fairfield KM et al^49^ (2019) | Maine Cancer Registry | Retrospective | Rurality | Age 20 - >75  Stage I or II  Stage III or IV  Small/Isolated Rural Large Rural Metropolitan | 2012-2015 | • Rurality was not associated with stage at diagnosis. • For early-stage disease (I or II), rurality was not associated with receipt of treatment. • For late-stage disease (III - IV), residents of large rural areas received more surgery (10%) compared with metropolitan (9%) or small/ isolated rural areas (6%), p=.01; received more chemotherapy (83%) compared with metropolitan (75%) or small/ isolated rural areas (79%). • After adjusting for covariates, patients in large rural areas received more chemotherapy (Odds Ratio 1.48; 95% Confidence Interval: 1.08-2.02), compared to patients in metropolitan areas. • Patients with early-stage disease residing in small/ isolated rural areas had delays in treatment (median time to first treatment=43 days [Interquartile Range (IQR), 22-68] compared with large rural (34 days, [IQR 17-55]), and metropolitan areas (35 days, [IQR 17-60]), p=.0009. |
| Treatment | Ferguson MK et al^50^ (2018) | Thoracic Surgery Directors Association located in Chicago provided list of thoracic surgeons and cardiothoracic trainees in academic medical centers | Prospective | Race of surgeon/trainees (White; Non-Hispanic; East Indian; East Asian; Black; White Hispanic; Middle Eastern; Other)  Race of standardized patient (SP) (Black; White) | Practicing surgeons  Trainees | Date of assessment not stated | • Surgeons were equally likely to recommend surgical interventions for Black and White standardized patients in contrast to clinical practice patterns that demonstrate Black patients undergo operations less often. |
| Treatment | Johnson AM et al^51^ (2020) | Florida Cancer Data System (FCDS), central cancer registry legislatively required to collect all cancer cases•  Florida Department of Health (DOH) responsible for administrating FCDS; Sylvester Comprehensive Cancer Center at the University of Miami Miller School of Medicine operates it | Retrospective | Race/Ethnicity, neighborhood segregation/deprivation | Age <50 - 64+  White  Black  Hispanic  Segregation/deprivation Low segregation, low deprivation  Low segregation, high deprivation  High segregation, low deprivation  High segregation, high deprivation | 2005-2014 | • Whites living in areas with high economic deprivation, regardless of level of Black or Hispanic segregation, associated with decreased odds of receiving treatment and surgery.  • Blacks living in highly segregated black areas, regardless of level of deprivation, associated with decreased odds of receiving treatment and surgery.  • For Hispanics, levels of segregation and deprivation not significant.  • Living in rural areas associated with increased risk of death for Whites and Blacks. |
| Treatment | Lutfi W et al^52^ (2020) | Registry - National Cancer Database | Retrospective | Race | Age  African American (AA) mean 66.2±10.7; Caucasian (CS) 69.4 ±10.3  African American  Caucasian | 2004-2015 | • AAs less likely to receive surgery (60.3% vs. 66.9%; p<.001) and more likely to receive external beam radiation therapy (EBRT) (12.4% vs.10.6%; p<.001) compared to CS.  • No significant difference in stereotactic ablative radiotherapy (SABR) utilization between races.  • After adjusting for covariates, AAs were less likely to receive definitive local therapy (surgery, SABR or EBRT) compared to CS, Odds Ratio: 0.64 (95% Confidence Interval [CI] 0.62–0.67) p<.001.  • From 2004 to 2015, surgery rates increased for AAs from 44.4% to 61.8% and for CS from 57.6% to 65.6%.  • AA patients had worse 5-year survival compared to CS (unadjusted analysis 46.7% vs. 47.9%; p = .009).  • After adjusting for treatment, AAs had improved survival (hazard ratio = 0.97, 95% CI = 0.94–0.99) compared to CS. |
| Treatment | Neroda P et al^53^ (2020) | Louisiana Tumor Registry (LTR), population-based  state cancer registry and a participant of the National Cancer Institute’s Surveillance, Epidemiology, and End Results (SEER)  program and the National Program of Cancer Registries of Centers for Disease Control and Prevention | Retrospective | Race, marital status, insurance, census tract level poverty, and census tract level urbanicity | Age <54-75+  White  Black | 2004-2016 | • Median time interval from diagnosis to surgery was 27 days in whites and 42 days in blacks.  • Overall, being unmarried, having Medicare or other public insurance, having Medicaid insurance, no insurance, and living in high poverty level area associated with a significantly higher likelihood of delayed surgery.  • Social support, private insurance, and living in census tracts with lower poverty level were associated with improved access to timely surgery.  • 28.7% of White and 48.4% of Black patients received delayed surgery.  • Black patients had almost two-fold odds of receiving delayed surgery than white patients.  • Census tract level poverty level a stronger effect on delayed surgery in Black patients than in White patients.  • Delayed surgery in Whites related to Medicare or no insurance coverage.  • Delayed surgery in Blacks related to Medicaid insurance. |
| Treatment | Nguyen DD et al^54^ (2021) | Registry - National Cancer Database (NCDB) | Retrospective | Race/Ethnicity | Age 40-64  Breast cancer  Colon cancer  Non–small-cell lung cancer Prostate cancer  White Non-Hispanic  Black Non-Hispanic  Hispanic  Asian American/Native Hawaiian/Pacific Islander  Non-Medicaid expansion states  vs Medicaid expanded states | 2009-2016 | • Receipt of definitive treatment for minorities in expansion states did not change compared with minority patients in non-expansion states.  • Compared to minorities in non-expansion states, the proportion of racial and ethnic minorities in expansion states receiving treatment within 30 days increased by 3.62%; 95% Confidence Interval [CI], (1.63 to 5.61), p<.001; no change at 90-days.  • Stratifying analysis by minority serving hospital status showed no effect on receipt of definitive therapy and time to treatment initiation at 30- and 90-days. |
| Treatment | Rapp JL et al^55^ (2020) | Registry - Surveillance, Epidemiology, and End Results database (November 2018 release)  North American Association of Central Cancer Registries (NAACCR) to identify “Reason for no Cancer-Directed Surgery” | Retrospective | Race/Ethnicity, sociodemographic | Age ≥18  Non-Hispanic (NH) Whites  NH Blacks  Hispanic  NH Others (Asian-Americans/Pacific Islanders/Alaskan-Natives/Native-Americans)  Recommended surgery  vs. not recommended surgery | 2007-2016 | • 95% recommended surgery underwent surgery and majority female (53.05%), within 65 to 79 years old (53.05%), non-Hispanic White (78.41%), with private insurance or Medicare coverage (69.66%), living within an urban county (86.46%) and married or living with a domestic partner (52.86%).  • Patients not recommended surgery were at increased risk of death compared with those who were recommended (propensity matched analysis). • Compared to NH Whites, NH Blacks and Hispanics had lower odds to receive surgery recommendation, Odds Ratio (OR) (95% Confidence Interval [CI]): 0.64 (0.59-0.70) and 0.75 (0.67-0.84), respectively.  • Effect of metro proximity was not significant. • Compared to Medicare, patients on Medicaid, non-specified insurance plan, and uninsured were less likely to be recommended surgery, OR (95% CI): 0.55 (0.51-0.60), 0.74 (0.69-0.79), and 0.45 (0.36-0.55), respectively. |
| Treatment | Stiles BM et a^56^ (2018) | Lung cancer database, not further specified (Weill Cornell Medicine, New York-Presbyterian Hospital; Institutional Review Board) | Retrospective | Race, Sex, Smoking status | Age Median (Interquartile Range [IQR]); Never Smoker 67 (58.8–75); Smoker 70 (62–76)  Never smoker; Smoker  White  Asian  African American Unknown/Others  Smoker  White  Asian  African American Unknown/Others | 1997-2016 | • Proportion of never smokers undergoing resection is increasing (16.1% in 1997-2006 vs. 26.6% in 2007-2016).  • Never smokers are more likely to be younger, female, Asian, and have adenocarcinoma histology, lower lobe tumors and pathological stage I disease.  • After propensity score matching, no difference in 5-year disease-free or cancer-specific survival between smokers vs. never smokers. |
| Treatment | Toubat O et al^57^ (2020) | National Cancer Database (NCBD), hospital-based oncology registry sponsored by the American College of  Surgeons and the American Cancer Society | Retrospective | Socioeconomic status (SES; race/ethnicity, median household income, education level, urban/rural area of residence, insurance status) | Age 18+  Race  White  Asian  Black  Hispanic  Other  Median Household income <$38,000  ≥$38,000  Urban  Rural | 2004-2014 | • 54.1% of patients received multiagent adjuvant chemotherapy (MAAC) after resection surgery.  • After adjustments rural patients were less likely to receive MAAC (Odds Ratio [OR], 1.23, p< .001).  • Patients with pN1 disease were less likely to receive MAAC if they resided in rural areas (OR, 1.23; 95% confidence interval [CI], 1.11-1.37; p<.001) or were uninsured or on Medicaid insurance (OR, 1.23; 95% CI, 1.07-1.41; p=.004).  • 5-year survival was significantly higher among those that received MAAC vs. surgery alone (53.6% vs 39.5%, log rank p< .001). |
| Treatment | Verma V et al^58^ (2019) | Registry - The National Cancer Database (NCDB), joint project of the Commission on Cancer (CoC) of American College of Surgeons and American Cancer Society | Retrospective | Race, insurance | Age ≥18  Caucasian  African American  Hispanic  Asian  Other  No Immunotherapy-type compound (IC) vs. Immunotherapy-type compound | 2004-2015 | • Temporal trends increased over time; 2004 to 2012, ≤1% received ICs; 4.9% in 2013, 6.6% in 2014, and 8.7% in 2015.  • ICs were more likely administered to younger and healthier patients, those living farther from treating facilities, and in more educated areas (p<0.05 for all).  • ICs were more often delivered to adenocarcinomas, and patients who received chemotherapy but not radiotherapy (p<0.05 for all).  • African Americans received less ICs compared to Caucasians, adjusted Odds Ratio (95% Confidence Interval): 0.869 (0.813-0.929), p<0.001.  • Uninsured and Medicaid populations received less ICs compared to other insurance types (p<0.05).  • African Americans were less likely to receive ICs even when stratified for Medicare, Medicaid, or private insurances. |
| Treatment | Maguire FB et al^59^ (2019) | California Cancer Registry (Data collected through network of regional  registries, affiliated with the National Cancer Institute's Surveillance Epidemiology and End Results (SEER) program | Retrospective | Health insurance | Age ≥ 20 y/o | 2012-2014 | • Overall, 51% of patients received systemic treatment, 32% did not, and 17% had unknown treatment status.  • More patients receiving systemic treatment had private insurance (58% vs. 48%) and fewer had dual Medicare–Medicaid or Medicaid/other public (29% vs. 35%) compared with the untreated group.  • Systemic treatment group had more Asian Pacific Islanders (19% vs. 12%) and fewer people in lowest neighborhood socioeconomic status quintile (14% vs. 20%).  • Significant disparities in receipt of any systemic treatment; receipt of bevacizumab; and receipt of tyrosine kinase inhibitors (TKIs) by source of health insurance after accounting for demographic and clinical factors.  • Blacks less likely to receive bevacizumab.  • Asians likely to receive TKIs. |
| Survival | Klugman M et al^60^ (2020) | Lung Cancer Clinical Cohort at Montefiore Medical Center Registry  Montefiore Medical Center/Einstein’s Cancer Registry  Electronic medical records | Prospective | Race/Ethnicity; Socioeconomic status, social support | Age >18 y/o  Non-Hispanic Whites  Hispanics/Latinos  Non-Hispanic Blacks  Non-Hispanic Asians | January 1, 2004-February 2017 | • Hispanic/Latino ethnicity compared to Non-Hispanic Whites was associated with a 15% decreased risk of death.  • Adjusted for clinical and social factors, Hispanic/Latino ethnicity compared to Non-Hispanic Whites was associated with a 30% decreased risk of death.  • Improved survival associated with surgery and non-smoking status.  • Strong sense of family (familismo) may contribute.  • Improved survival in Hispanics/Latinos may be due to differences in environmental exposures, culture, tumor genetics, and/or lung cancer therapy. |
| Survival | Klugman M et al^61^ (2019) | Meta-Analysis (Literature Search) | Retrospective | Race/Ethnicity | Age not reported  27 U.S. studies 9 reported Hispanics; 24 reported African Americans; 11 reported Asian Americans  All studies accounted for age, race and/or ethnicity, smoking status; stage  Majority of studies accounted for socioeconomic status (N = 16), Histology (N = 19)  Nearly all studies accounted for treatment (Radiation, surgery and/or chemotherapy; N = 23) | Articles published through 2018  Individual cumulative study periods include 1990-2013 | • After adjusting for clinical factors and smoking status, Hispanics and Asians experienced improved survival compared to Non-Hispanic Whites.  • No significant difference in survival between African American and White race after adjustment.  • Prognostic factors were female gender (Hazard Ratio [HR] = 0.88, 95% Confidence Interval [CI] 0.87–0.89), unmarried status (HR = 1.08, 95% CI 1.04–1.11), ever-smoking status (HR = 1.11, 95% CI 1.08–1.15), having comorbidities (HR = 1.39, 95% CI 1.24–1.56), and treatment receipt (surgery: HR = 0.33, 95% CI 0.32–0.34; radiation: HR = 0.87, 95% CI 0.85–0.88; chemotherapy: HR = 0.64, 95% CI 0.63–0.65). |
| Survival | Jones CC et al^62^ (2018) | Southern Community Cohort Study linkage with State Cancer Registries in 12 Southern states (Alabama, Arkansas, Florida, Georgia, Kentucky, Louisiana, Mississippi, North Carolina, South Carolina, Tennessee, Virginia, and West Virginia) | Prospective | Race/Ethnicity,  African ancestry | Age 40-79    Self-reported Blacks  Self-reported Whites | March 2002-September 2010 | • Median percentage of African Ancestry for Blacks 85.6% and Whites 1.3%.  • African ancestry was not associated with overall survival.  • Stage and treatment are robust predictors of lung cancer survival. |
| Smoking | Bekalu MA et al^63^ (2019) | *Click to Connect (C2C)* randomized control trial designed to examine media and internet behaviors of urban poor with particular interest in health  Survey instrument used in C2C was adapted from standard and validated measures that are publicly available from the US Centers for Disease Control and Prevention and the US National Cancer Institutes’ Health Information National Trends Survey | Retrospective | Socioeconomic status | Age 25-60  Education  Annual household income | Data collection period not specified | • Likert scale (1=Strongly Agree, 2=Agree, 3=Disagree, and 4=Strongly Disagree).  • Disagreement with statement smoking cigarettes has not been proven to cause cancer was significantly higher among individuals with smoking experience than those without.  • Agreement with statement inhaling someone else’s cigarette smoke can cause lung cancer in non-smokers was marginally higher in smokers than non-smokers.  • After controlling for covariates, results indicated that health-related interpersonal communication was positively associated with beliefs about the link between smoking and lung cancer risks. |
| Hospice | Johnson LA et al^64^ (2019) | Electronic Health Records from large tertiary cancer center and regional satellite cancer treatment sites located in the Southeastern U.S.  2013 National Center for Health Statistics Urban-Rural Classification Scheme for Counties | Retrospective | Race, rurality | Age 25 to ≥85  Black  White  Others  Rural  Urban  Advanced directive  No  Yes | January 1, 2015-June 30, 2016 | • Hospice care N= 86 (36%). • Older patients longer hospice stays (8.0 days, 35-44.9 y/o; 5.8 days, 55-64.9 y/o; 11.4 days, 65-74.9 y/o; 19.3 days, 75-84.9 y/o, and 25.7 days, ≥85 y/o). • Patients without an advanced directive less likely to enroll in hospice. • No significant disparities in hospice utilization or hospice length of stay for race, age, or rural/urban area. |
| Occupational Risks | Juon HS et al^65^ (2021) | National Lung Screening Trial (NLST) | Retrospective | Race/Ethnicity, occupational exposure risks | Age 55 - 75  Non-Hispanic Whites  Non-Hispanic African Americans  Others (Asian, Native Hawaiian or Pacific Islander, American Indian, Hispanic, or more than one race)  Current or former (quit within past 15 years) smoker with at least a 30 pack-year history | NLST study period | • Overall NLST lung cancer rate 3.9%; African Americans (4.3%) higher rate of lung cancer diagnosis than Whites (3.9%) and Others (2.9%).  • African Americans (31.8%) higher rate of occupational exposure than Whites (27.9%).  • About 28% (NSLT) reported at least one occupational exposure, including 6.5% exposed to silica and 4.7% to asbestos.  • African Americans (9.9%) higher rate of silica exposures than Whites (6.3%).  • Others (7.1%) and African Americans (6.9%) had higher rates of asbestos exposure than Whites (4.5%).  • After adjusting for covariates, African Americans had higher odds of lung cancer diagnosis than Whites (Odds Ratio [OR] = 1.24 to 1.25, 95% Confidence Interval [CI] = 1.01-1.54).  • Current smokers with occupational exposures had higher odds of lung cancer diagnosis compared to those without lung cancer (adjusted OR = 2.01, 95% CI = 1.76-2.30).  • Current smokers with exposure to silica (adjusted OR = 2.35, 95% CI = 1.89-2.91) or asbestos (adjusted OR = 1.97, 95% CI = 1.52-2.56) had higher odds of lung cancer. |
| Follow-up After Positive Screening | Sesti J et al^66^  (2020) | National Lung Screening Trial (NLST) | Retrospective | Race, sociodemographics | Age 55 - 75  White  Black or African American  Asian  American Indian or Alaskan Native  Native Hawaiian or other Pacific Islander  More than one race  Positive lung screening test. Follow-up vs. No follow-up | 2002-2004 | • Women had a statistically higher follow-up rate compared to men (90% vs 88.8%, p≤ 0.05).  • Patients reported as married or living as married had higher rate of follow-up compared to those who were not (90.2% vs 87.5%, p≤ 0.05).  • African American patients had significantly lower follow-up proportion compared to White patients (82.8% vs. 89.6%, p≤ 0.05).  • Education level was not a significant factor in follow-up rates.  • Current smokers followed up at lower rates compared to former smokers (87.9 % vs 90.6%, p≤ 0.05).  • Logistic regression determined sex, marital status, race, and smoking status to be predictors of follow-up. |
